# Hyper-Eosinophilic Syndrome Masquerading as Myocardial Infarction, Stroke and Cancer

**DOI:** 10.7759/cureus.9630

**Published:** 2020-08-09

**Authors:** Arun Minupuri, Karthik Ramireddy, Roshni Patel, Samia Hossain, Jesus Salas Noain

**Affiliations:** 1 Internal Medicine, Mercy Catholic Medical Center, Darby, USA

**Keywords:** hyper-eosinophilia syndrome, blood disorder, end-organ-damage, steroids

## Abstract

Hyper-eosinophilic syndrome (HES) can be fatal if left untreated; and it is difficult to make a diagnosis early on due to the symptoms overlapping with many other conditions. For patients presenting with eosinophilia and end-organ damage, clinicians should have a high degree of suspicion for HES. Treatment with steroids can prevent further progression or can lead to complete resolution of the symptoms.

## Introduction

Hyper-eosinophilic syndrome (HES) comes under the category of rare blood disorders, where eosinophils invade the blood vessels, causing multi-organ failure. Diagnosis is made by an absolute eosinophil count >1500 cells/microL, associated organ damage, and exclusion of other known causes of eosinophilia [1]. Symptoms depend on organ dysfunction and may include skin rashes, shortness of breath, chest pain/discomfort, abdominal pain/nausea/vomiting, or behavioral changes [2]. These symptoms are common in many other medical problems, which can impede making an initial diagnosis [3].

The prevalence of HES is unknown, and further research is yet to be done to understand these disorders [4]. HES initially was considered to be idiopathic, but recent advances have paved the way for subclassification. It can be divided into three categories: primary/neoplastic, secondary/reactive, and idiopathic [5]. We present a case report of an individual who presented with signs and symptoms of multi-organ failure, and after exclusion of other diagnoses came to the conclusion of the idiopathic hyper-eosinophilic syndrome.

## Case presentation

A 68-year-old African-American female presented to our institution with complaints of swallowing difficulties, decreased appetite, and generalized weakness for one week prior to admission. Swallowing has been progressively getting worse, initially started with solid foods, and then progressed to liquids, along with the association of pain leading to decreased by mouth intake. She denied any fevers/chills, nausea/vomiting, abdominal pain, or diarrhea/constipation. Review of systems elicited chest discomfort for two days prior to admission, along with shortness of breath on exertion. Additionally, endorsed weight loss of 80-100 pounds over a one-year timespan.

Her past medical history included hypertension, diabetes mellitus type 2, and asthma. Surgical history included cholecystectomy. She was a social drinker and had 30 pack-years of smoking history. Medications included hydrochlorothiazide, amlodipine, and metformin. She denied any allergies.

Upon arrival at the emergency department, she was hemodynamically stable with a stable airway and without fever. Labs were concerning for a white blood cell count of 36.2 Thou/uL, with a differential neutrophil 29%, lymphocytes 16%, and eosinophils 52% (absolute eosinophil count: 18.82 Thou/uL).

**Figure 1:**
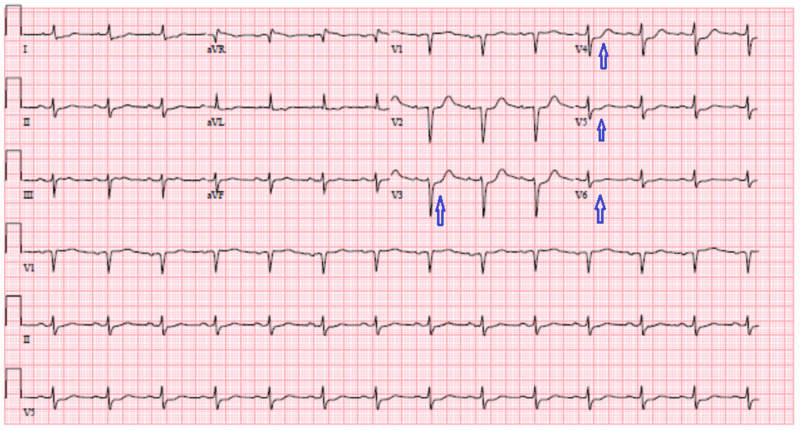
EKG 1-2 mm ST depressions in lateral leads V3-V6 (blue arrows).

Other derangements included a troponin of 6.73 ng/mL, which continued to trend upwards to reach a peak of 13.17 ng/mL. Initial chest imaging was negative for any consolidation. Figure 1 shows EKG 1-2 mm ST depressions in lateral leads V3-V6.

The patient was started on a heparin drip along with aspirin and Plavix® and was admitted to the telemetry floor.

**Figure 2:**
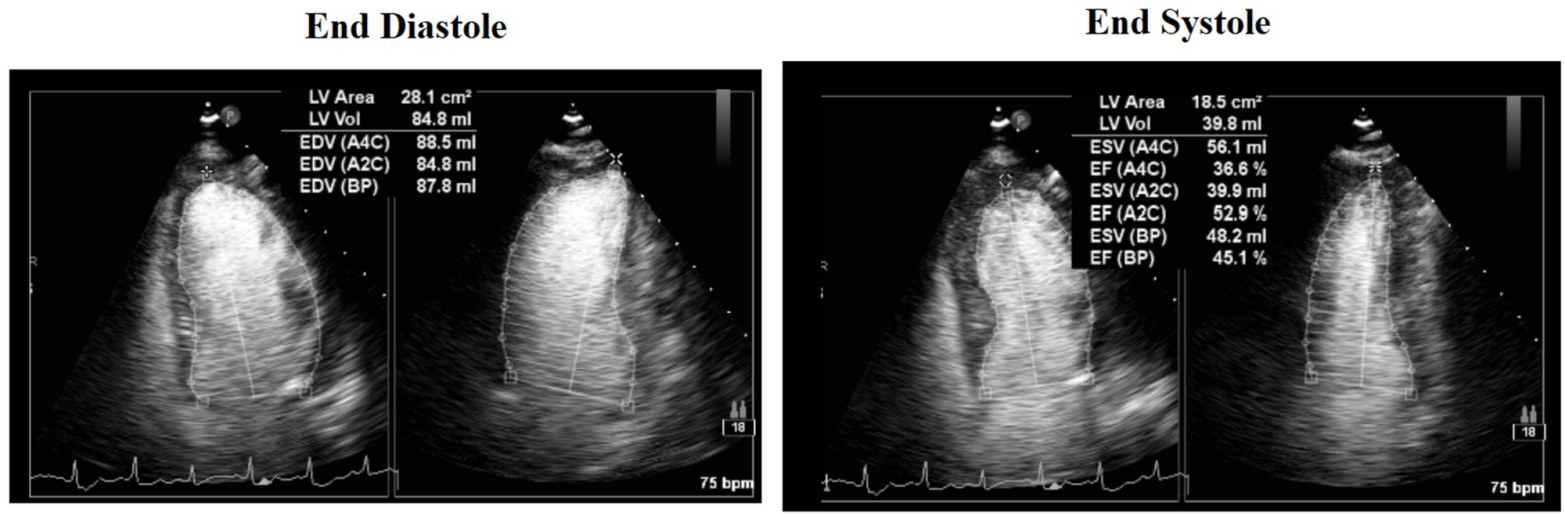
Cardiac echocardiogram End diastole versus end systole views: ejection fraction of 37%, moderate global hypokinesis.

**Figure 3:**
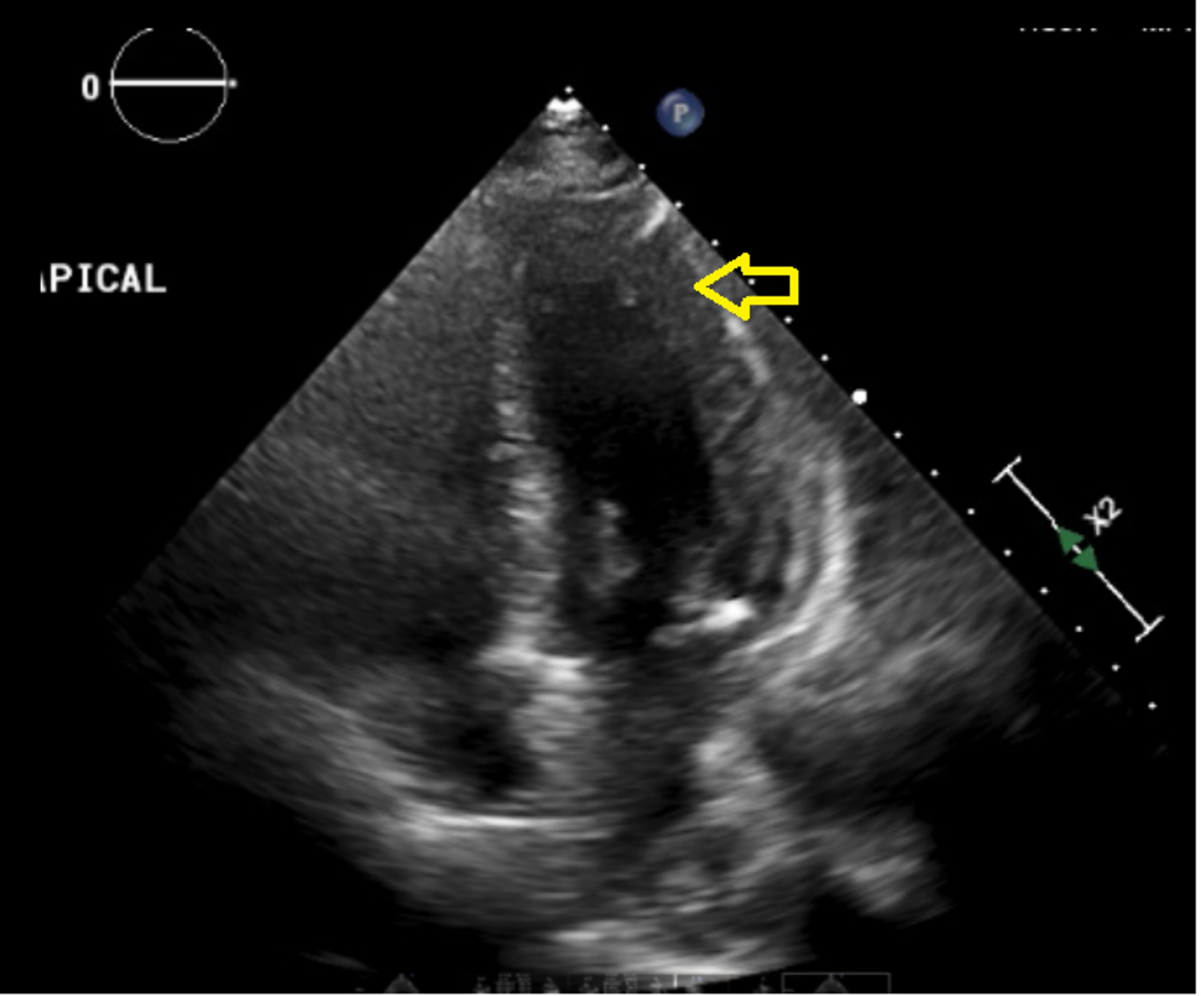
Cardiac echocardiogram Apical view: moderate concentric left ventricular hypertrophy (yellow arrow).

Echocardiogram revealed moderate concentric left ventricular hypertrophy, moderate global hypokinesis of the left ventricle, with an ejection fraction of 37% (Figures 2-3). She underwent left heart catheterization with findings of single branch vessel coronary artery disease that was not amenable to percutaneous coronary intervention.

**Figure 4:**
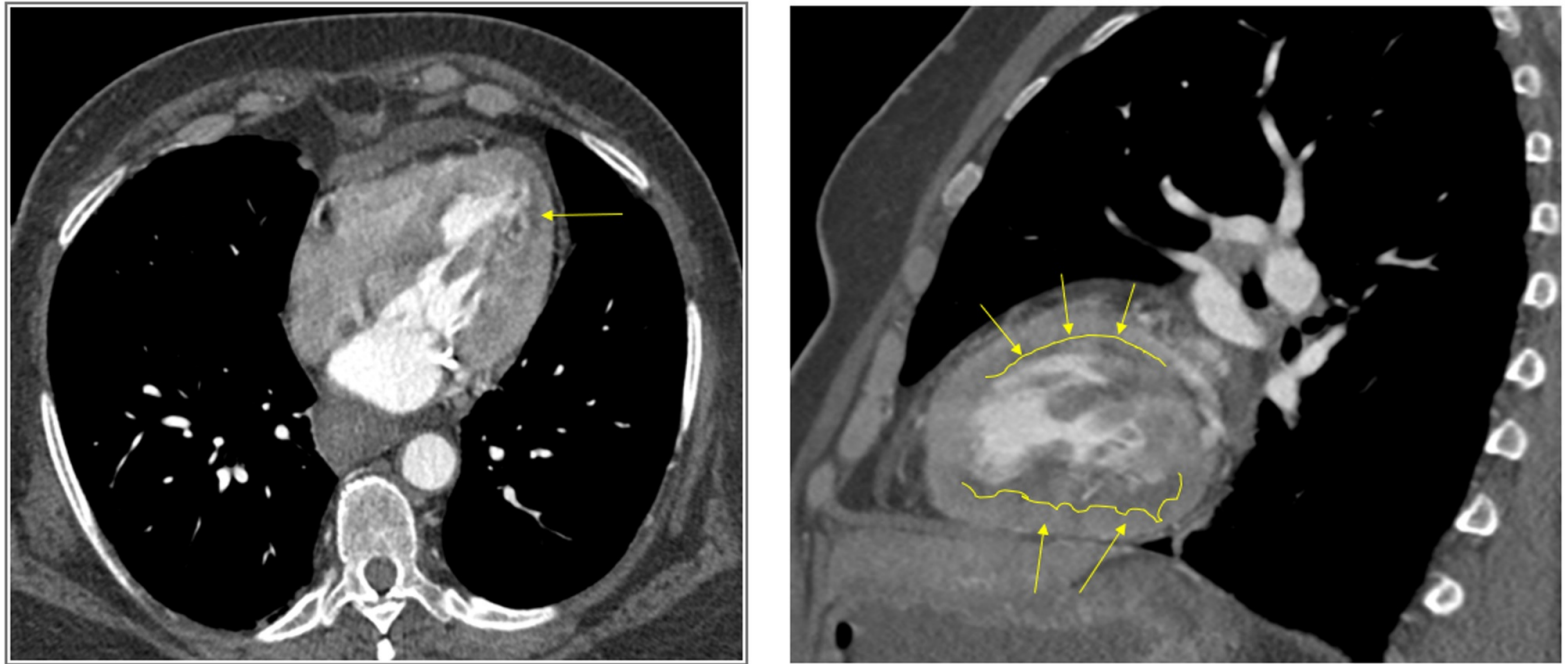
Cardiac MRI Subendocardial hypoperfusion, along with delayed enhancement in multiple vascular territories involving the left ventricular wall and papillary muscle in keeping with ischemia/infarction (yellow arrows).

Cardiac magnetic resonance imaging (MRI) was ordered due to coronary artery disease out of proportion to her cardiomyopathy and clinical symptoms and a strong suspicion of myocarditis. Findings revealed subendocardial hypoperfusion, along with delayed enhancement in multiple vascular territories involving the left ventricular wall and papillary muscle in keeping with ischemia/infarction (Figure 4).

**Figure 5:**
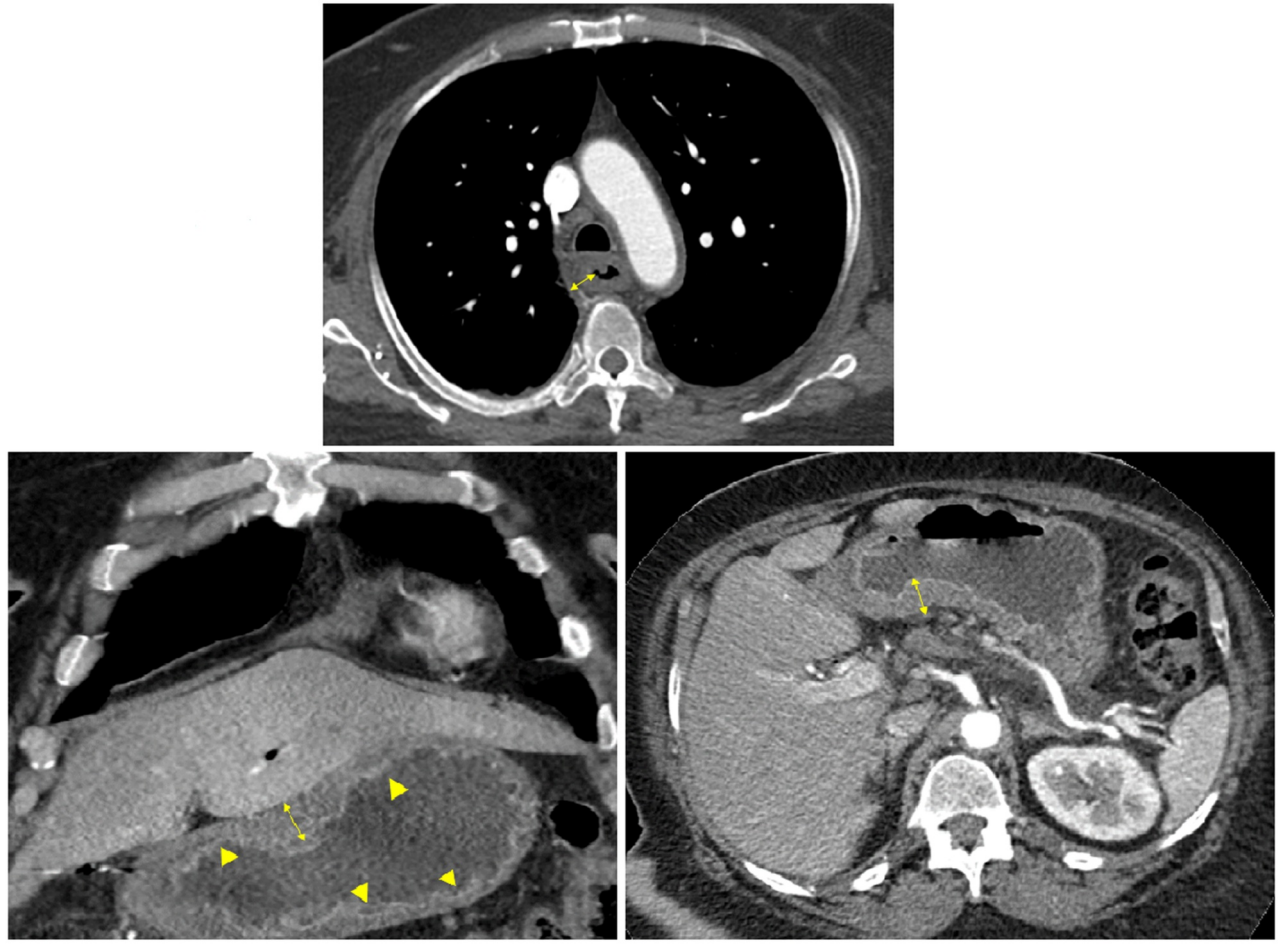
CT of chest and abdomen Upper image: long segment circumferential thickening of the esophagus representing esophagitis (yellow double arrows). Lower images: diffuse gastric wall thickening and mucosal enhancement representing gastritis (yellow arrowheads, and double arrows).

**Figure 6:**
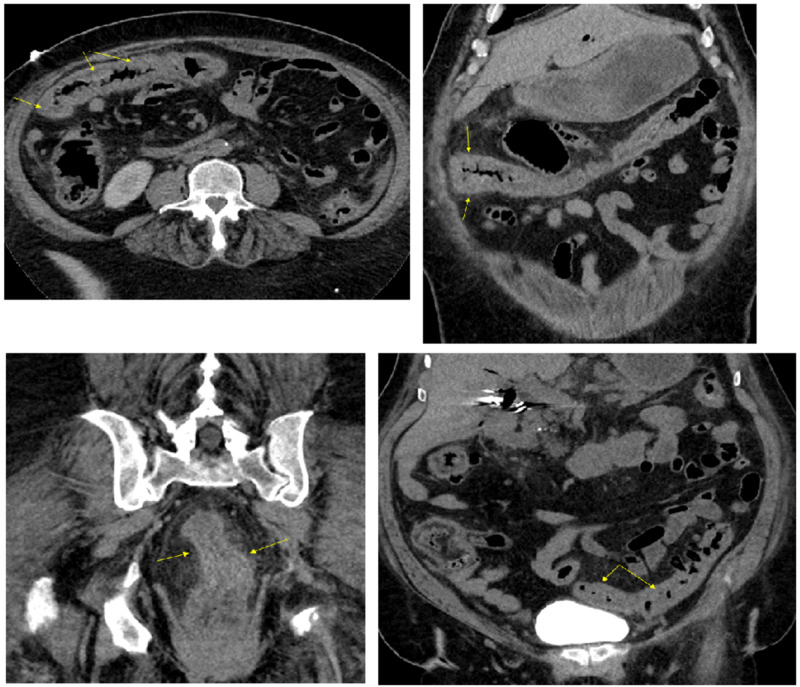
CT of abdomen and pelvis Diffuse mucosal thickening of colon with pericolic stranding suggestive of pancolitis (yellow arrows).

Additional imaging in the form of computed tomography (CT) chest /abdomen/pelvis with/without contrast revealed- a long segment of circumferential thickening of the esophagus, thickening of the stomach, diffuse mucosal thickening of the colon with pericolic stranding, suggestive of severe esophagitis, gastritis, and colitis (Figures 5-6). CT of the brain was negative for any acute findings.

Peripheral smear revealed eosinophilia, but no abnormal cells. Stool studies were negative.

**Figure 7:**
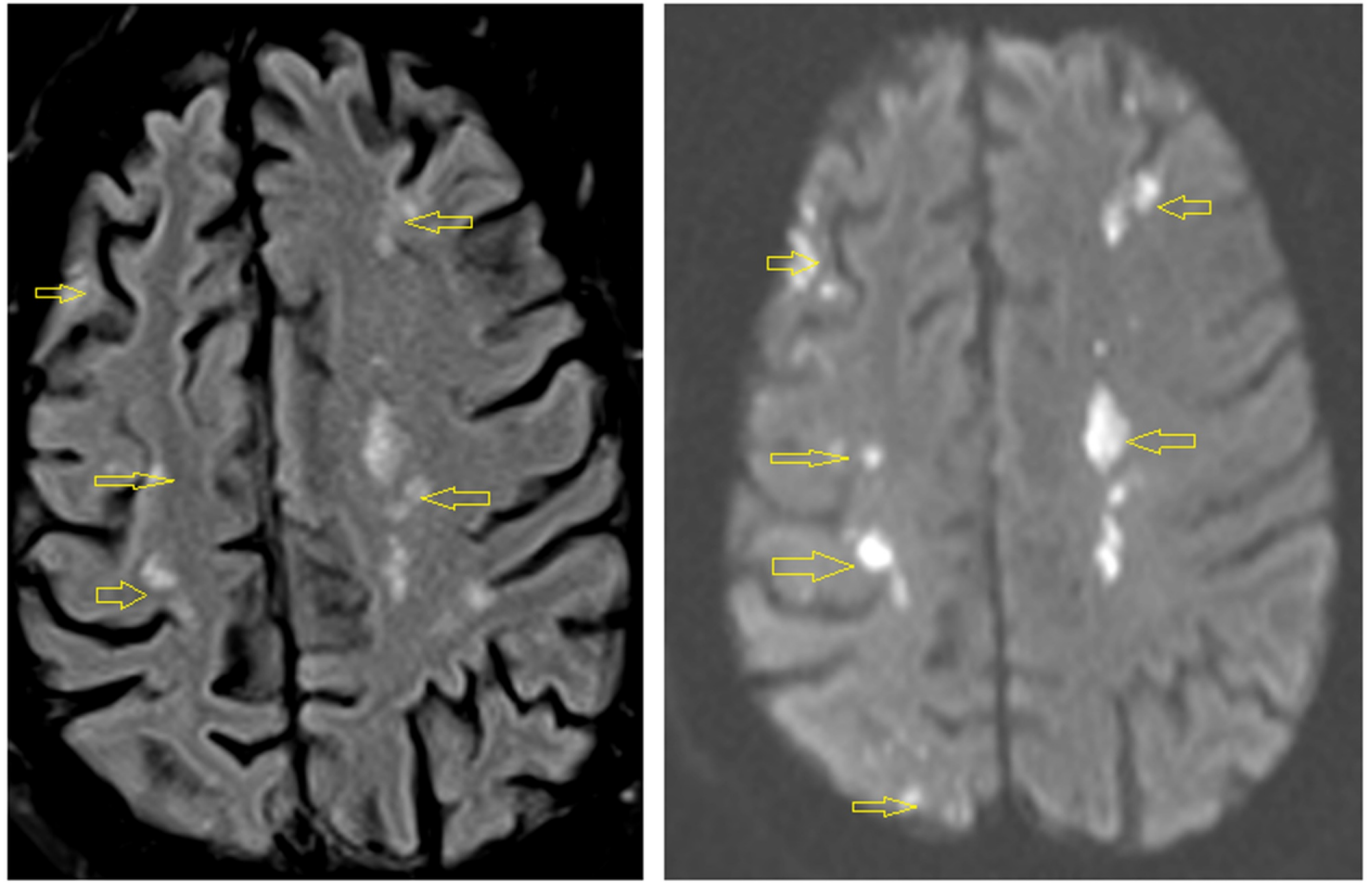
Brain MRI Multiple areas of restricted diffusion throughout the hemispheres and posterior fossa in multiple vascular distributions, suggestive of multiple emboli with acute/subacute infarctions (yellow arrows).

On the third day of admission, the patient complained about left upper extremity weakness. MRI of the brain was done, which revealed multiple areas of restricted diffusion throughout the hemispheres and posterior fossa in multiple vascular distributions, suggestive of multiple emboli with acute/subacute infarctions (Figure 7).

On the fourth day of admission, the patient underwent endoscopy, which revealed normal esophagus, gastritis, and normal duodenum.

**Figure 8:**
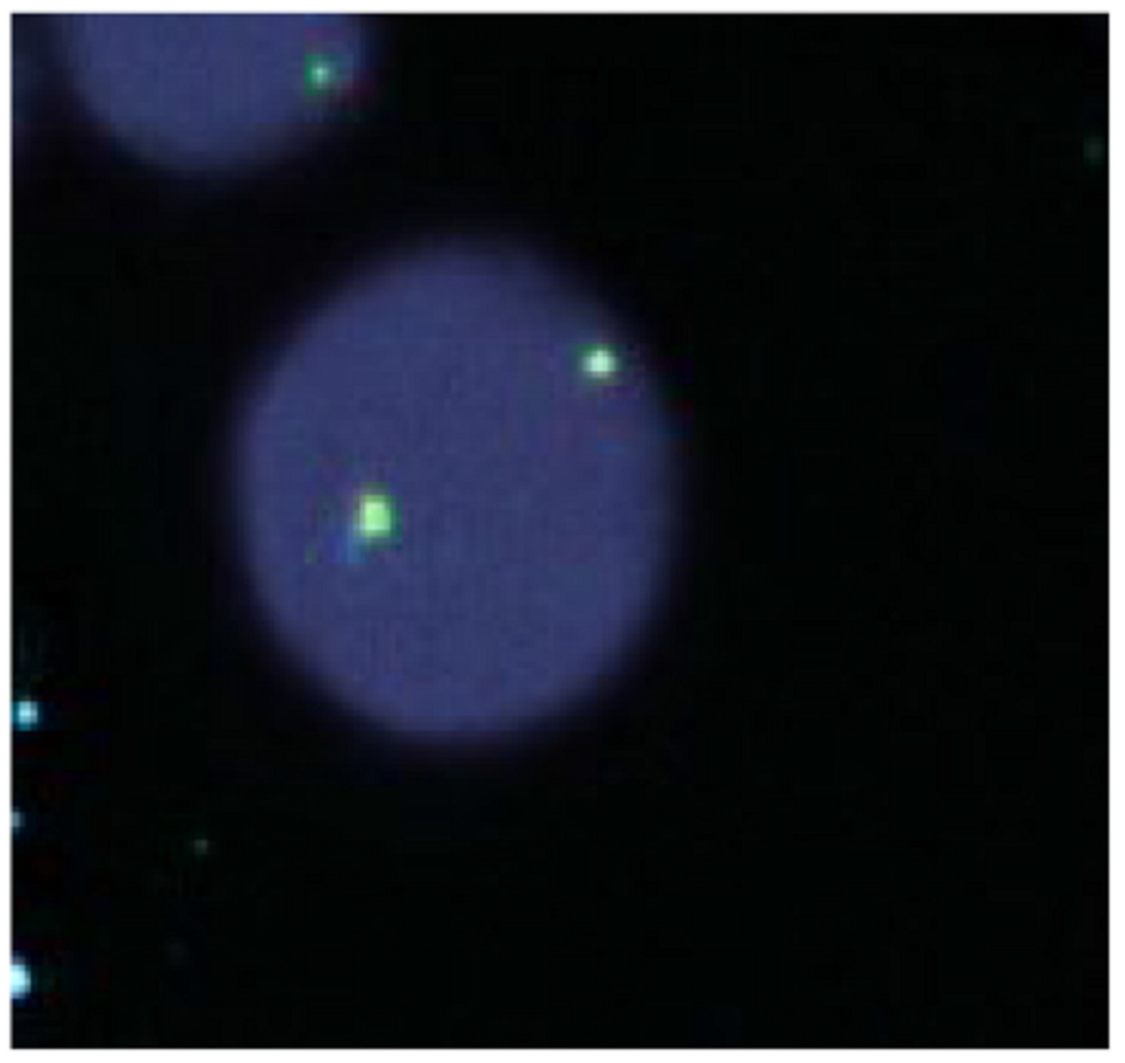
FISH analysis Fluorescent in-situ hybridization (FISH) was negative for lymphoid or myeloid neoplasms with eosinophilia.

Bone marrow studies were done, which included biopsy, cytology, cytogenetics, and gene-rearrangement. Findings included: hypercellular bone marrow with a marked increase in eosinophils; flow cytometry: approximately 45% eosinophils without an increase in blasts, or an immunophenotypically abnormal lymphoid population; no other increase in immunophenotypic abnormalities; fluorescent in-situ hybridization was negative for lymphoid or myeloid neoplasms with eosinophilia (Figure 8); JAK2, V617F mutations were not detected.

Vasculitis work-up was negative with normal anti-nuclear antibody, C- anti-neutrophil cytoplasmic autoantibody (ANCA), P-ANCA, and complement levels.

Steroids were initiated 60mg/day, as part of the treatment for hyper-eosinophilic syndrome, and the patient showed clinical signs of improvement. She was discharged in stable condition, and repeat blood work in two months revealed a white blood cell count of 10.5 Thou/uL, differential neutrophils 64%, lymphocytes 28.4%, eosinophils 2.6% (absolute eosinophil count 0.27 Thou/uL).

## Discussion

HES can be difficult to diagnose as the symptoms it produces are associated with a variety of other disorders [3]. Patients presenting with eosinophilia will need a complete workup ruling out allergic disorders, infections, vascular-related disorders, malignancy, drug-related, and gastrointestinal/pulmonary related conditions [2].

In our case, the patient presented with a multitude of initial symptoms that included dysphagia, odynophagia, chest discomfort, shortness of breath, and weight loss. This coupled with elevated leukocytes with eosinophilia and elevated troponins on admission along with EKG changes led to the differential diagnosis of myocardial infarction and underlying malignancy. Cardiac catheterization, CT of chest/abdomen/pelvis, endoscopy, and bone marrow studies being negative led to the above diagnosis less likely. Stool studies in the form of ova parasites, stool gram stain and culture being negative have ruled out gastrointestinal related infections. 

Even though cardiac catheterization was negative, suspicion of underlying cardiac pathology in the form of myocarditis remained high. Cardiac MRI was ordered for confirmation, but instead, it revealed subendocardial hypoperfusion due to ischemia/infarction. With these findings and the patient developing cerebral infarctions in varying vascular territories, there was a concern for vasculitis. After vascular studies turned out to be unrevealing, we led to the conclusion of HES.

It is essential to characterize the subtype of HES, as the treatment varies with each. The primary subtype is due to underlying stem cell, myeloid or eosinophil neoplasm, whereas secondary subtype is related to overproduction of cytokines which can be seen in parasitic infections, certain solid tumors and T cell dysfunction [6]. Our case does not belong to either the primary or secondary, therefore it fits into the subtype of idiopathic HES where the underlying cause remains unknown despite adequate workup.

The cornerstone treatment for HES consists of 1mg/kg/day or 60mg/day of steroids, but treatment with tyrosine kinase inhibitors and monoclonal antibodies are emerging [7-9]. Mechanism of glucocorticoids in HES is unclear, but potential theories include: inhibiting formation of new eosinophils, apoptosis or sequestration of eosinophils [10]. After starting our patient on methylprednisolone there was noted the improvement in her clinical symptoms, and she was stable to be discharged to the sub-acute nursing facility with oral steroids at a tapered dose. Repeat blood work two months later showed complete resolution of eosinophilia.

HES is a rare disorder that can have a high mortality if left untreated, and our case emphasizes the difficulty in obtaining an accurate early diagnosis [11]. As research continues to evolve, we may have better understanding of the pathophysiology and at newer treatments in tacking this fatal disease. For now, clinicians should be mindful of the broad range of diagnosis that eosinophilia represents; and initiating therapy early on with steroids for a high suspicion of HES.

## Conclusions

Clinicians need to be vigilant on the broad differential of peripheral eosinophilia, and should always consider HES when it leads to end-organ damage. It can be a fatal disorder, but starting treatment early on with steroids can slow the progression or can lead to complete resolution.
